# Progress in State Medicine

**Published:** 1904-01-30

**Authors:** 


### Progress in State Medicine.

### CREMATION.

Blair,[1] in reviewing the progress made in cremation in this country, mentions that his experience in removing 1,100 bodies from 700 square acres of land, for a street improvement, convinced him that from a public health point of view cremation is far preferable to earth interment. At the International Congress of Hygiene held in London in 1891, the subject of cremation was brought forward by M. Georges Salomon, general secretary of the Cremation Society of France, advocating the general adoption of cremation. A resolution to the effect that "the cremation of the dead is a rational and hygienic procedure which is specially called for where death occurs from contagious disease," was carried by an overwhelming majority, there being only four dissentients. The first crematorium in England was opened by the Cremation Society of England at Woking in 1885, and the number of bodies cremated annually increased continuously up to 1900, during which year 301 operations took place, the total up to the end of 1902 being 2,119. In 1892 a crematorium was opened near Manchester, followed by one in Glasgow in 1895, and one in Liverpool in 1896. So far these crematoria had been started by private companies, but under the provision of local Acts, the corporations of Hull and Darlington opened crematoria in 1901, and under similar powers the Corporation of Leicester also completed a suitable establishment in 1902. In addition to the crematorium at Golder's Green, near London, the City Corporation of London have also taken up the subject, and the construction of a crematorium at the City of London Cemetery at Ilford is about to be commenced under powers obtained by private Bill in 1900. The number of cremations carried out at these establishments during the last ten years has slowly but steadily increased, being 131 in 1893, and 446 in 1902. This progress is slow if compared with other countries. In the United States there are 25 establishments, at which about 2,700 cremations per annum take place; in Italy there are 27 crematoria, 7 in Germany, 2 in France, 3 in Switzerland, 2 in Sweden, and 1 in Denmark. In 1902 Parliament passed the Cremation Act, which applies to England and Scotland, and authorises any burial board, council, or other local authority having powers and duties of the burial board, and any local authority maintaining a cemetery, to provide a crematorium to be used under regulations made by the Secretary of State. This Act came into operation on the first day of April of this year. The committee appointed to draw up these regulations made special inquiry into the question of cremation being used to destroy evidence of crime, and in the report admitted that whilst no regulations could be formed which would entirely eliminate the risk, yet they concluded that regulations could be framed which would so far reduce the risk as to make cremation at least as safe as the existing method of burial, as in certain circumstances the law even now permits burial without certification of the cause of death. Sentiment offers the greatest hindrance to the more general adoption of the process of cremation. Two great advantages over earth burial are (1) greater safeguarding of the health of the living, and (2) the absolute destruction of the germs of contagious diseases, which is rendered perfectly certain where the human remains are destroyed at a temperature of 1,800° to 2,000° Fahr., now attained in cremator furnaces. The resuscitation of diseases by disturbance of the ground where many years before diseased bodies had been buried, has been proved over and over again, and even without intentional disturbance of such ground there is always the possibility of germs being brought to the surface and dispersed in the air by natural action, such as that of earth-worms. So far as this country is concerned there are only two types of furnace adopted, namely, the "Gorini" furnaces at Woking, and a modification of the same, but not differing much, at Liverpool; and the "Simon" furnace, which has been constructed at all the other crematoria in this country. The "Gorini" variety consists of a furnace about 4 feet by 2 feet 6 inches, in which a coke fire is maintained, and in direct continuation of this furnace, separated only by a firebrick bridge, is an incinerating chamber, through which the gases of the furnace pass on all sides of the coffin, passing away from this downwards to a flue leading to the chimney. The whole structure of the furnace is about 16 feet long by 7 feet wide and 8 feet high from the floor. The furnace and incineration chamber and flue are lined with firebricks, but the structure is contained within solid walls strengthened by brick-stays and tie-bolts. The "Simon" furnace is a much more complicated structure; is about 14 feet long by 10 feet wide and about 16 feet high, involving the construction of a basement story. The furnace is placed at the base of the structure, and its gases pass into a large chamber in the centre of the block, and from this by five flues on either side, into a smaller chamber immediately below the incinerating chamber. Into this they have access not only through the perforated bottom, but through side passages, where there is admitted a quantity of air heated by its passage through the flues immediately in contact with the central chamber, and capable of regulation as to volume, the object being to secure increased temperature by regenerative combustion in the incineration chamber. From this the gases pass away through a perforated arch, and by a series of downcast flues on each side to the basement, where they converge into one flue leading to the shaft. In all cases the chimney shaft is placed at some little distance from the furnace, so as to be clear of the main buildings. In order to secure a sufficient draught when first starting the fire it is found an advantage to have what is called a pilot fire at the base of the chimney. It is generally found, except at the first lighting of the fires, that there is practically no visible smoke during the process of cremation, and as the design of the shaft generally takes the form of an ornamental tower, it appears quite as an architectural feature rather than a plain chimney stack. The cost of the establishment at Woking was £5,000. About two hours is necessary from the lighting of the fires to obtain the necessary heat, and a further two hours is occupied in the actual incineration process, about one ton of coke being necessary for the whole operation, the charge for which is £5. At Manchester the furnace chamber is at the back of the chapel, and contains two furnaces, one behind the other, which proves to be an inconvenient arrangement, as it is necessary, in using the most distant furnace from the chapel, to pass the coffin through the furnace chamber of the nearer furnace, which of course, must be cold at such a time. The cost of constructing the whole of the buildings, but apart from the cost of land, was about £7,000. The charge for cremation is about £5 5s., but with a view to encourage the operation among those who cannot afford so much, a reduced charge of £2 2s. is made. The Glasgow crematorium differs from all others in this country in two particulars: firstly, instead of the coffin sliding horizontally from the catafalque into the furnace chamber, the catafalque is lowered vertically into vaults below; and secondly, the furnace is heated by gas produced on the premises from small coal. The production of the gas takes place in a separate apparatus from the incinerating chamber, where it reaches a temperature of 1,800° Fahr. The charges for cremation vary from £2 2s. to £5 5s. At Hull the charges are to parishioners £1 1s. and to non-parishioners £3 3s., the charge in the former barely covering cost of fuel and labour. With regard to cremation in India,[2] it is reported that "contrary to official expectation appreciable quantities of arsenic were easily detected in the ashes and bones from the scene of cremation. The loose regulations with regard to the disposal of the dead in India favour the crime of poisoning by arsenic very considerably, but the imperfect cremation of the body as generally practised would appear to hold out promise for the detection of the criminals." It is pointed out that when organic matters are incinerated in the presence of arsenic, the alkaline salts which are formed combine with the poison and fix it in the shape of non-volatile arsenite or arsenate. The alkaline salts of the body formed on burning would be sufficient to fix the otherwise volatile arsenic and to convert it into a stable and non-volatile salt. The crematorium[3] recently erected at Golder's Green, just beyond Hampstead Heath, has been for some time in working order, and already 116 bodies have been cremated there. There are two furnaces fed by coke, and the actual consumption of the body is brought about by overheated air, the temperature within the cremation chamber reaching about 2,000° Fahr. A columbarium has been provided in an attached building, which will afford space for about 2,000 urns. With regard to a proposed crematorium for Leeds, Harrison[4] stated to a Local Government Board Inspector that the proposed site was 200 yards away from the nearest dwelling-house and 50 yards from the nearest highway. Instead of the usual coke furnace it was proposed to use a new method of incineration by gas. There would be absolutely no smell from the new crematorium, and the chimney was to be encased by a very elaborate tower, so that it would not be an eyesore. This new system would be less expensive to users of the crematorium than was the case under the coke system. The estimated cost was about £2,500. The crematorium[5] which is to be erected by the Bradford Corporation in Scholemoor Cemetery, will cost about £5,000. Gas furnaces will be used, being more cleanly and less liable to cause smoke. Further, the temperature can be more easily regulated, whilst the low price of gas in the city will considerably lighten the cost. The heat of the furnace will not exceed 1,500° Fahr., although in some crematoria the temperature reaches 2,000°, but it has been found that abnormally high temperatures do not give such satisfactory results. The furnace will be ready for cremation as soon as the interior glows with a bright orange colour. The gases from the furnace will be conducted to the chimney, which is encased in a massive masonry tower. The latter will be in keeping with the ecclesiastical character of the building and its surroundings, and from the external appearance there will be no suggestion of the presence of a chimney.

[1] Pub. Health Engin., June 27, 1903. [2] Lancet, Aug. 15, 1903. [3] Brit. Med. Jour., Oct. 3, 1903. [4] Pub. Health Engin., Nov. 7, 1903. [5] Ibid., Nov. 28, 1903.

